# Effects of Electrical Pulse Width and Output Irradiance on Intense Pulse Light Inactivation

**DOI:** 10.3390/bioengineering9120730

**Published:** 2022-11-26

**Authors:** Shuge Xie, Diya Shen, Qing Yuan, Zairui Wu, Junyuan Wang, Fusheng Li, Qiuyi Han, Shanduan Zhang

**Affiliations:** 1 Institute for Electric Light Sources, Fudan University, 2005 Songhu Road, Shanghai 200438, China; 2 Ningbo Fotile Kitchen Ware Co., Ltd., 218 Binhai 2nd Road, Ningbo 315336, China

**Keywords:** intense pulse light (IPL), inactivation efficiency, pulse width, irradiance, spectral action

## Abstract

The effects of electrical pulse width and output irradiance on the inactivation effect of intense pulse light (IPL) are studied in this paper. The measured radiant efficiency of pulsed xenon lamp can be more than 50%, and its irradiance can reach levels 100-times greater than that of a low-pressure mercury lamp. *Staphylococcus aureus* is used in inactivation experiments. When the irradiance and dose are both constant, there is no significant difference in inactivation efficiency when the pulse width is changed. However, a narrow pulse width corresponding to high irradiance at the same single-pulsed dose displays better inactivation effect. Experimental results are compared between the xenon lamp and low-pressure mercury lamp. The reduction factor (RF) value of the xenon lamp is more than 1.0 higher under the condition of both the same dose and irradiance. In order to achieve the same RF value, the dose of continuous-wave light must be at least three-times greater than that of pulsed light. The spectral action of the pulsed light is also studied. It is confirmed that UVC plays a major role across the whole spectrum. The experimental results show that extreme high-pulsed irradiance presents the main contributing factor behind the excellent bactericidal effect of IPL.

## 1. Introduction

Intense pulse light (IPL) can release extremely high peak-power radiation in a very short time, and has been adopted in inactivation technology [1]. It bears many advantages such as high penetration, real-time control, safety and so on [2]. Currently, IPL is widely used in water purification [3], the food industry [4,5] and various other applications. The IPL generator is composed of a pulsed light source (usually pulsed xenon lamp), capacitor, boost and trigger module [6].

The inactivation mechanism of IPL includes genetic material destruction by ultraviolet (UV) irradiation [7,8], as well as protein degradation through thermal effects of visible and infrared bands [9]. In addition, pulse effect with transient high energy has a significant impact in cellular component damage.

The crucial inactivation effect of IPL has been validated [10]. The use of 64 light pulses with 1 μs pulse width can kill 99.9% of *Escherichia coli* and 99% of *Listeria* [11]. The number of *Staphylococcus aureus* bacteria decreased by 7–8 orders of magnitude after receiving IPL of 5.6 J cm^−2^ [12]. IPL at a dose of 13.05 J cm^−2^ inactivates *facultative anaerobes* by 2.91 orders of magnitude, and 4.30 J cm^−2^ inactivates *murine norovirus* by 3.35 orders of magnitude [13]. IPL of 7.40 J cm^−2^ can reduce the number of *Bacillus subtilis* spores by 7 orders of magnitude [14]. A 100-s pulsed ultraviolet light treatment of *Aspergillus niger* spores could reduce its number by 4.93 orders of magnitude [15].

The effect of various electrical parameters has been studied on the inactivation efficiency of IPL, since they determine the characteristics of the light and are easily tested. It is indicated that frequency, duty cycle and input power are the most important parameters regarding inactivation ability [16,17,18]. The inactivation effect of pulsed light is better than that of continuous-wave (CW) light under the same input electrical power, and the superiority increases with the fluence [19]. As for optical parameters, irradiance (distance) and irradiation dose (treatment) also have great impacts [20,21,22]. A decrease in distance can increase the irradiance, and an increase in treatment can increase the irradiation dose. High irradiance or irradiation dose can promote the inactivation effect of IPL conspicuously [23]. However, increasing the pulse width reduces the irradiance and radiation dose, thereby weakening the inactivation effect [24]. In addition, the wavelength plays a decisive role in the inactivation process. Studies have shown that the inactivation rate at 270 nm was the highest for *Escherichia Coli*. No significant inactivation effect was observed for wavelengths greater than 300 nm. This trend is consistent with the spectral absorption curve of DNA [25,26]. According to the inactivation curves of ultraviolet and visible bands, the inactivation effect of UV irradiation produces much better results than that of visible light [27,28].

So far, there has been extensive research on IPL inactivation. However, the dependence of pulse width or duty cycle on the inactivation effect has not been studied under the conditions of constant irradiance or radiation dose. Although the effect of the wavelength used has been studied, most of the microorganisms studied were *Escherichia coli* and only specific wavelengths were selected. At the same time, the irradiation dose represents the main parameter of concern in the research process while the irradiance is ignored. In addition, the irradiation dose is directly equivalent to the fluence obtained through dividing the energy by the irradiated area, which produces insufficiently accurate results.

In this paper, the photoelectric parameters of pulsed xenon lamp were measured and evaluated. Subsequently, the effect of pulse width was studied excluding the influence of irradiance or IPL dose. Further, the high-irradiance superiority of IPL was confirmed by a quantitative comparison of narrow-band pulsed light and CW light at a central wavelength of 254 nm, which provides a theoretical basis for its wider application. In addition, the narrow-band IPL inactivation effects of light from the UV–visible band were studied under the same irradiance and dose.

## 2. Materials and Methods

### 2.1. Experimental Setup

The tested pulsed xenon lamp (Fotile, Ningbo, China) had a total length of 200 mm and a diameter of 18.0 mm. It was driven by a customized power module (LASERPWR QPM500-SA-V1, Jinan, China). Mixed Domain Oscilloscope (TEKTRONIX MDO3034, Beaverton, USA) was used to measure the values of transient voltage *u*(*t*) and current *i*(*t*) of the lamp. A spectrometer (Instrument Systems, CAS140CT, Munich, Germany) was used to measure the spectra. The irradiance *E*_1_ was measured by an energy meter (THORLABS PM100D, Newton, MA, USA), which produces a response within the wavelength range of 185–25,000 nm. According to the accuracy of the test instruments, the uncertainty of these physical parameters is less than 5%.

### 2.2. Calculation of Photoelectric Parameters

The record length of oscilloscope was 10,000 points with a sample interval of 0.1 μs. The transient power *p(t)* can be obtained by multiplying *u*(*t*) and *i*(*t*) at each point. Effective power *P*_elec_ was calculated with Equation (1),

(1) $$P_{\mathrm{elec}}=\frac{\sum p\left(t\right)∆t}{T}$$

where *T* refers to a complete pulse period. Single-pulsed electric power *P*_sp_ was calculated in the following equation where *f* refers to the frequency. In addition, FWHM refers to the width corresponding to half of the maximum current, which was regarded as the electric pulse width of pulsed xenon lamp approximately.

(2) $$P_{\mathrm{sp}}=\frac{\;P_{\mathrm{elec}}}{f}$$

The pulsed xenon lamp can be regarded as a linear light source, so the radiant flux (radiant power) *P*_r1_ can be calculated using the Keitz formula [29], as shown in Equation (3), in which *E*_1_ is the measured irradiance, *L* is the length of the lamp, *D* is the distance between the lamp axis and the probe of the energy meter, and the half apex angle *α* can be derived in Equation (4). Subsequently, the radiant efficiency *η* can be obtained using Equation (5).

(3) $$P_{r1}=\frac{{2\pi }^{2}DL}{2\alpha +\sin2\alpha }E_{1}$$

(4) $${\alpha =\tan}^{-1}\frac{L}{2\;\times \;D}$$

(5) $$\eta =\frac{\;P_{r1}}{P_{\mathrm{sp}}}\;\times \;100\%$$

### 2.3. Inactivation Experimental Setup

The pulsed power supply was customized to adjust the parameters in the inactivation experiments. It can set a variety of parameters including electric pulse width and frequency. All the inactivation experiments were operated in a shading box (500 mm × 250 mm × 500 mm) composed of stainless steel. Since the duty cycle of pulsed light was not 100%, the irradiation dose *Q* should be calculated using Equation (6), in which *τ* denotes pulse width, *N* represents flash times, and *Q*_1_ denotes single-pulsed irradiation dose. Theoretically, *τ* should be the optical pulse width, which was approximately replaced by the electrical pulse width in this study. In addition, the frequency was uniformly set to 1 Hz for the whole experiment, so that the levels of single-pulsed electric power and effective power were the same. As a result, the radiant efficiency can be the radiant power divided by the single-pulsed electric power.

(6) $${Q=\tau \;\times \;E}_{1}{\;\times \;N=Q}_{1}\times \;N$$

Two kinds of experimental setups were adopted to evaluate the dependence of pulsed width on the inactivation efficiency. First, the irradiance was kept constant at the same distance between the lamp and the working surface. The same irradiation dose was ensured through adjusting the flash times, and the pulse width was set between 40 to 180 μs with a gradient of 20 μs. Second, the single-pulsed irradiation dose was kept equal with constant flash times by adjusting the irradiance. In the experiment, the pulse width was set from 40 to 120 μs.

When quantitatively comparing the inactivation efficiency of pulsed light and CW light, pulsed xenon lamp with band-pass filter (254 ± 5 nm) was used to achieve a similar radiation wavelength as the low-pressure mercury lamp (GMY UVCH36H17X411-2G11). The two lamps were both approximately linear light sources, and placed into the shading box to ensure the same irradiation effect. The inactivation ability was compared by the inactivation efficiency under the same irradiation dose, as well as by the irradiation dose required to achieve the same inactivation efficiency.

**Figure 1:**
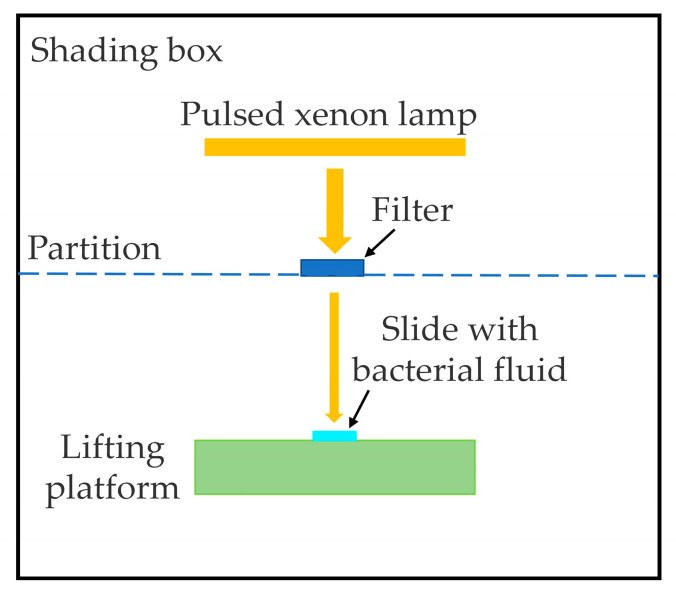
Schematic diagram of the experimental setup.

In addition, the influence of IPL wavelength was also studied by using various band-pass filters (EDMUND OPTICS, 2 inches in diameter). A shading plate was placed under the lamp with a hole that was distanced 30 mm from the axis of the lamp tube, as shown in Figure 1. The filters could be placed upon the hole, which could only transmit light of specific wavelengths. The spectral action of various radiation bands on the inactivation were tested and evaluated, including ultraviolet and visible light. During the experiment, uniformity of the irradiation dose and the irradiance were ensured.

### 2.4. Inactivation Experiment Process

All the inactivation experiments were operated in a sterile environment. The microorganism used was *Staphylococcus aureus* (CMCC (B) 26003). A total of 4–6 *Staphylococcus aureus* generations were often chosen, because its number could increase by 4–5 orders of magnitude and the performance was relatively stable. The first step was coating the bacterial fluid (10 μL) by the pipette gun on the glass slide (10 mm × 10 mm) evenly. The thickness of bacterial fluid was 100 μm calculated by dividing the volume by the bottom area. After the slides were naturally air dried, the thickness measured less than 20 μm by micrometer. Next, the control group was directly eluted, diluted and cultured. Notably, the eluent was Phosphate Buffered Saline (PBS), the diluent was Tryptone Physiological Solution (TPS), and the culture medium was Tryptic Soy Agar (TSA). After being irradiated by IPL under different conditions, the experimental group was treated by the same follow-up operations as those of the control group. Both groups were then cultured in a 37 ℃ incubator. After 18–24 h, an effective range between 30–300 was selected for counting the number of bacteria with a colony counter. It should be noted that each group experiment was repeated three times and the average value was taken.

The inactivation efficiency was measured by calculating the reduction factor (RF) in Equation (7), where *N*_0_ is the number of the control group and *N*_1_ is that of the experimental group. RF = 1 denotes that the inactivation rate was 90%, RF = 2 means the inactivation rate was 99%, and so on. The greater the RF value, the higher the inactivation efficiency [30]. In this study, the error bar for all experiments was adopted as *μ* ± *σ*, in which *μ* represents the sample mean and *σ* is the standard deviation.

(7) $${\mathrm{RF}=\log}_{10}\frac{N_{0}}{N_{1}}$$

## 3. Results and Discussion

### 3.1. Photoelectric Parameters of Pulsed Xenon Lamp

The photoelectric parameters corresponding to a pulse width between 40–120 μs are shown in Table 1. The single-pulsed electric power increased significantly when the pulse width increased, but the peak power *P*_max_ was enhanced slightly. The peak power was above 300 kW with a duration of about 100 μs, which indicated that the pulsed light had an extremely high instantaneous peak energy. For the optical parameters, the irradiance also increased significantly with an increase in pulse width. It is worth noting that the radiant efficiency stayed basically unchanged, namely above 50%. High radiant efficiency allows the pulsed xenon lamp to achieve a higher radiant flux under the same input conditions, so as to shorten the inactivation time. The measured irradiance of low-pressure mercury lamp was 120 μW cm^−2^. Compared to this most commonly used UV light source in activation, the pulsed xenon lamp achieved an irradiance 100-times higher. In addition, the measured FWHM was the same as the set pulse width value, demonstrating the accuracy of the power supply parameter settings. 

**Figure 2:**
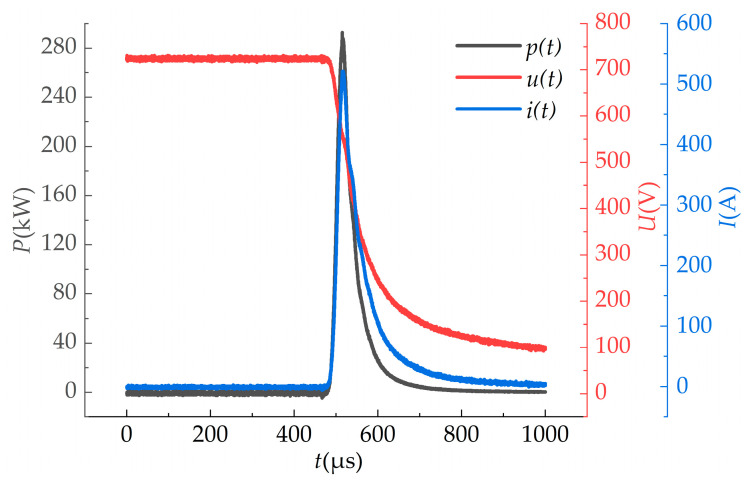
Oscillograms of voltage, current and power.

The transient current, voltage and electrical power of the IPL with a pulse width of 40 μs are shown in Figure 2. It is clear that the pulsed xenon lamp achieved an extremely high peak power exceeding 280 kW over a short period of time.

**Figure 3:**
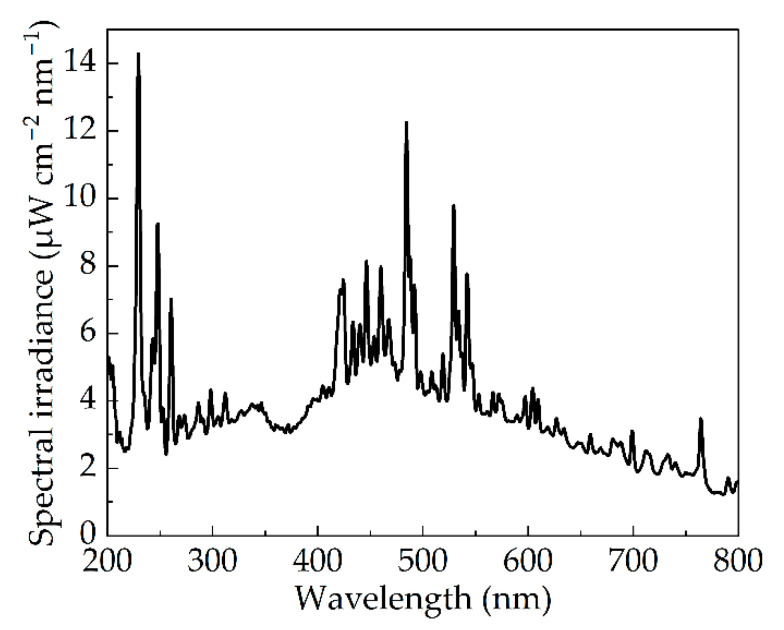
Instantaneous spectral distribution of pulsed xenon lamp.

The instantaneous radiation spectrum of the xenon lamp is shown in Figure 3. The results showed that the pulsed xenon lamp had a wide spectral range. According to the theory of gas discharge [31], the spectrum of pulsed xenon lamp should be composed of spectral lines and continuous spectrum. The former was generated by electron energy level transition, and the latter was mainly generated by recombination. The energy level transition can occur between any energy levels of adjacent atoms, so the linear spectrum irradiance can be observed across all wavelengths. The intensity of linear spectrum was different due to the different number of electrons in the transition. The UV peak wavelength of the xenon lamp in this paper was 229.2 nm, whose corresponding spectral irradiance was 14.3 μW cm^−2^ nm^−1^. In the visible range, the peak wavelength was 484.0 nm with a spectral irradiance of 12.7 μW cm^−2^ nm^−1^. The peak spectral irradiance of the visible band reached almost 86% that of the ultraviolet band.

The irradiance of each spectral band was equal to the integral of the spectral irradiance in the range of the corresponding wavelength. So the radiation percentage of each band to the whole spectrum could be evaluated, as shown in Table 2. Compared with other pulsed xenon lamps [32], the percentage of this work’s UV irradiance was basically the same. However, the percentage of UVC radiation in the experiment was almost twice that of other lamps, the percentage of UVB was roughly the same, and the percentage of UVA was reduced by 40%. Considering that UVC plays a major role in microorganism inactivation by pulsed light [26], the pulsed xenon lamp used in the work was expected to have higher inactivation efficiency given that the power input remains constant.

### 3.2. Effect of Pulse Width on Inactivation Efficiency

**Figure 4:**
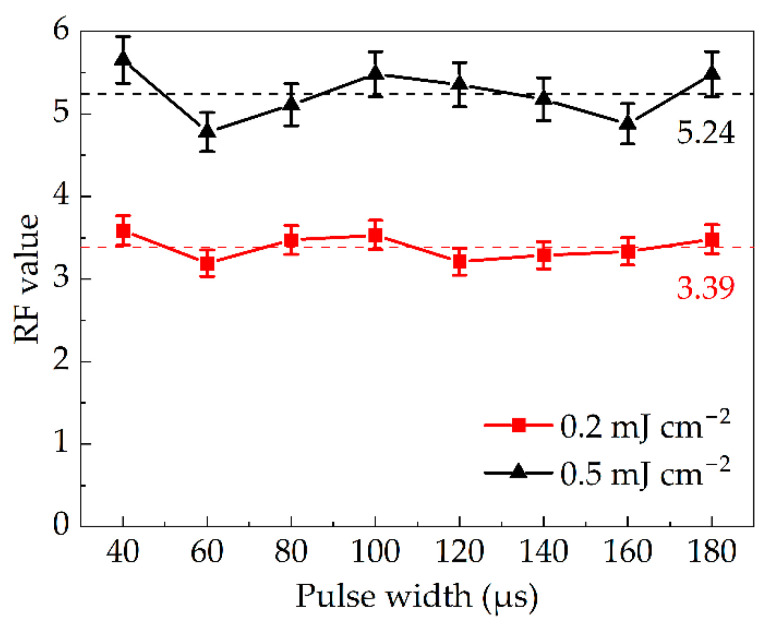
Dependence of RF value on pulse width.

The curve of the RF value corresponding to pulse width is shown in Figure 4 under the conditions of both constant irradiance and dose. The irradiance was determined to be 35.0 mW cm^−2^. When the irradiation dose was set at 0.2 mJ cm^−2^, the required flash times were 32, 36, 41, 48, 57, 71, 95 and 143, and a pulsed width between 40 and 180 μs with a gradient of 20 μs. At this time, for all pulse widths the RF value was >3.0. When the dose increased to 0.5 mJ cm^−2^, the corresponding flash times were 79, 89, 102, 119, 143, 178, 238 and 357. Here, the overall RF value reached more than 4.5. For these two curves, the coefficient of variation (CV) can be calculated using Equation (8) [33], giving 0.0456 and 0.0587, respectively. The experimental results showed that when both the irradiation dose and irradiance were the same, the pulse width had no significant effect on the inactivation efficiency within the allowable error range. A reasonable explanation was that when the dose and irradiance were the same, only the pulse width affected flash times. However, the flash times could have an influence on the inactivation effect, only if the dose was changed.

(8) $$\mathrm{CV}=\frac{\sigma }{\mu }$$

**Figure 5:**
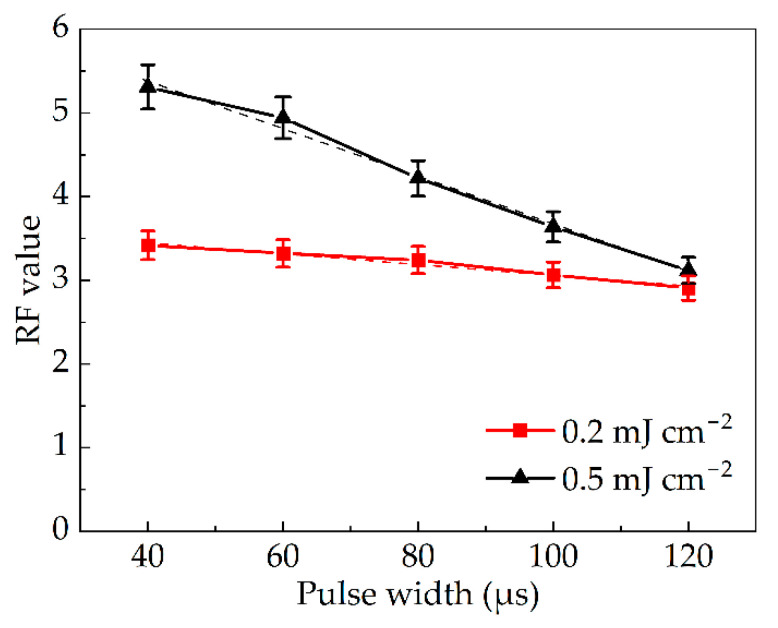
Dependence of RF value on pulse width at the same single-pulsed dose.

In the second experiment, the single-pulsed dose was set constant at 1.44 μJ cm^−2^. The irradiance corresponding to a pulse width of 40, 60, 80, 100 and 120 μs is 36.0, 24.0, 18.0, 14.4 and 12.0 mW cm^−2^. As shown in Figure 5, the RF value decreases from 3.4 to 2.9 with an increase in pulse width when the total irradiation dose is 0.2 mJ cm^−2^. When the dose is increased to 0.5 mJ cm^−2^, the downward trend in RF value becomes more conspicuous, decreasing by two orders of magnitude from 5.3 to 3.1. Linear trend predictions of the two curves are shown in Equations (9) and (10) with pulse width as the independent variable and RF value as the dependent variable. *R²* was generated with the equation automatically and represented the fitting degree of linear prediction. The higher the value (close to 1.00), the better the fitting effect. The slopes of the two equations were both negative with *R²* equaled 0.975 and 0.993, indicating that the inactivation efficiency was negatively correlated with the pulse width when the irradiation dose of total and single-pulsed remained unchanged. The larger the pulse width, the lower the inactivation efficiency. This is because at the same single-pulsed dose, the irradiance corresponding to narrower pulse width is higher. Even if the total dose was equal, the instantaneous peak energy corresponding to narrow pulse width is higher, causing a more destructive effect to *Staphylococcus aureus.* Increasing the pulse width weakened the inactivation effect when both the total and single-pulsed doses were kept unchanged. Increasing the pulse width also reduced the inactivation rate when the input voltage was constant [24].

(9) $$0.2\;\mathrm{mJ}\;{\mathrm{cm}}^{-2}\;\mathrm{dose}:\;{\mathrm{RF}}_{1}=-0.0064\tau +3.70,\;R_{1}^{2}=0.975$$

(10) $$0.5\;\mathrm{mJ}\;{\mathrm{cm}}^{-2}\;\mathrm{dose}:\;{\mathrm{RF}}_{2}=-0.0284\tau +6.52,\;R_{2}^{2}=0.993$$

For industrial applications, it is difficult to adjust the irradiance due to the fixed nature of the equipment. Thus, the pulse width should be kept as small as possible in order to achieve a greater inactivation effect, based on the technological capacity of the power supply.

### 3.3. Comparison of Inactivation Effects between Pulsed Light and Continuous Light

**Figure 6:**
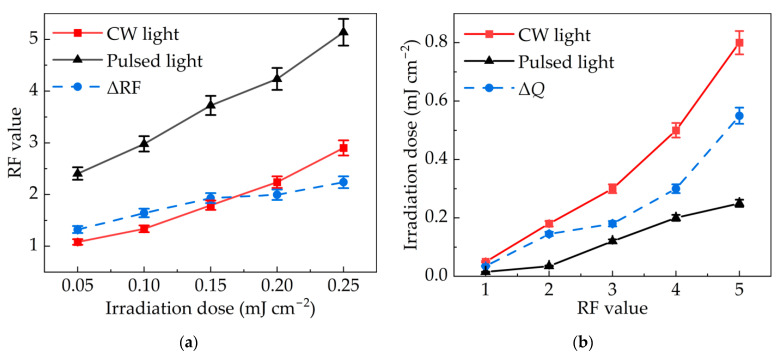
Comparison of inactivation effects between pulsed light and CW light: (**a**) RF value under the same irradiation dose and (**b**) dose required under the same RF value.

The superiority of IPL inactivation can be preliminarily extrapolated according to the tendency indicated in Figure 5. This result is further confirmed by the comparison between IPL and CW light. The RF value of the pulsed xenon lamp with a pulse width of 120 μs was compared to that of the low-pressure mercury lamp at the same irradiation dose in Figure 6a. The doses were set at 0.05, 0.10, 0.15, 0.20 and 0.25 mJ cm^−2^. The irradiance of the CW light was 6.75 μW cm^−2^, corresponding to exposure durations of 7, 15, 22, 30 and 37 s. With the pulse width set at 120 μs, the irradiance of the pulsed xenon lamp was 1.125 mW cm^−2^. So the flash times were 370, 741, 1111, 1481 and 1852. When the dose increased from 0.05 mJ cm^−2^ to 0.25 mJ cm^−2^, the RF value of the low-pressure mercury lamp increased from 1.1 to 2.9, while that of the pulsed xenon lamp increased from 2.4 to 5.1. At the same dose, the RF value of pulsed light was more than 1.0 higher than that of CW light throughout the entire experimentation period. This meant it could achieve higher inactivation efficiency under the same irradiation dose. This is because the instantaneous high-intensity radiation of pulsed light had a stronger impact. Even if the doses were the same, the inactivation effect would still be more powerful. Moreover, the experimental results also indicate that the RF value of the combination of pulsed xenon lamp and filter was 4.2 when the dose was 0.20 mJ cm^−2^. Compared with the above experiment, the average RF value of full-spectrum xenon lamp at the same dose was only 3.4, as shown in Figure 4. Additionally, it was proved that UVC was the main band for inactivation.

In order to intuitively show the inactivation advantages of IPL, the irradiation doses required by the two lamps when realizing the same RF value were directly compared in Figure 6b. Through repeated experiments, the irradiation dose was determined by constantly adjusting irradiation time and flash times. The dose required for CW light was at least twice that of pulsed light to achieve the same RF value. Furthermore, the larger the RF value, the faster the dose required for CW light increased. This meant it was more difficult for the low-pressure mercury lamp to achieve higher inactivation requirements.

The difference in RF value (ΔRF) between pulsed light and CW light under the same irradiation dose, and the difference in required dose (Δ*Q*) to reach the same RF value were supplemented in Figure 6a,b. After linearly fitting the two curves as shown in Equations (11) and (12), it was found that ΔRF was positively related to the irradiation dose. With the increase in dose, although the RF values of the two modes of light increased, the growth rate of pulsed light was significantly greater than that of CW light. The experimental results showed that the inactivation effect of pulsed light on *Staphylococcus aureus* was greater than that of CW light when the same irradiation dose was applied. On the other hand, to achieve the same RF value for *Staphylococcus aureus*, Δ*Q* of the two modes also increased with an increase in RF value. This means the higher the inactivation requirement, the greater the dose required for CW light.

(11) $$\Delta \mathrm{RF}=4.36Q+1.17,\;R_{1}^{2}=0.960.$$

(12) $$\Delta Q=0.12\mathrm{RF}-0.11,\;R_{2}^{2}=0.910$$

Next, the influence of irradiance on inactivation effect was avoided by producing the same average irradiance per second. The average irradiance per second of pulsed light *E* was calculated as 0.135 μW cm^−2^ in Equation (13). Regarding the low-pressure mercury lamp, this could be realized by adding a neutral density filter (THORLABS, 2 inches in diameter) with a transmittance of 2%. In this case, the irradiation dose was the same as long as the irradiation time was equal. The dose was kept unchanged, and the corresponding exposure durations were 370, 741, 1111, 1481 and 1852 s.

(13) $$E=\frac{{\tau \times f\times E}_{1}}{T}\;$$

**Figure 7:**
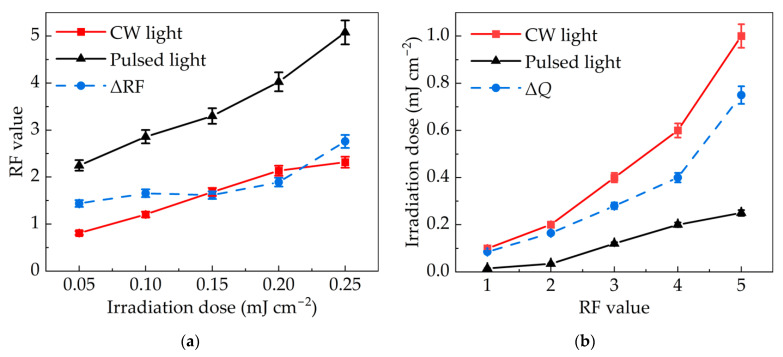
Comparison of inactivation effects between pulsed light and CW light with the same average irradiance per second: (**a**) RF value under the same irradiation dose and (**b**) dose required under the same RF.

Figure 7a shows the RF value–irradiation dose curves, in which the difference between the CW light and pulsed light increased 7(a). The main reason is that the decreased irradiance of low-pressure mercury lamp weakened its inactivation efficiency.

The irradiation dose required for CW light and pulsed light to reach the same RF value was also compared when the average irradiance was the same. Here, the dose required for CW light was at least three-times that of pulsed light (Figure 7b). The increased dose of the low-pressure mercury lamp was also to compensate for the losses in reduced irradiance.

Under the condition of controlling the average irradiation per second, two difference curves were also added in Figure 7a,b. The results showed that ΔRF and Δ*Q* were much greater after removing the influence of exposure duration. The average ΔRF increased from 1.83 to 1.87 and the average Δ*Q* increased from 0.24 to 0.34. At the same time, the slopes of the two linear equations are larger as indicated in Equations (14) and (15), demonstrating that the inactivation advantage of pulsed light was more conspicuous when the exposure duration was the same.

(14) $$\mathrm{Same}\;\mathrm{average}\;\mathrm{irradiance}\;\mathrm{per}\;\mathrm{second}:\;\Delta \mathrm{RF}=5.76Q+1.01,\;R_{1}^{2}=0.761$$

(15) $$\mathrm{Same}\;\mathrm{average}\;\mathrm{irradiance}\;\mathrm{per}\;\mathrm{second}:\;\Delta Q=0.16\mathrm{RF}-0.13,\;R_{1}^{2}=0.904$$

The results in this paper are consistent with the conclusion derived by the comparison results using tunable frequency power supply, which is that the inactivation rate of pulsed light at different frequencies was always higher than that of CW light [16].

### 3.4. Effect of Wavelength on Inactivation Efficiency

**Figure 8:**
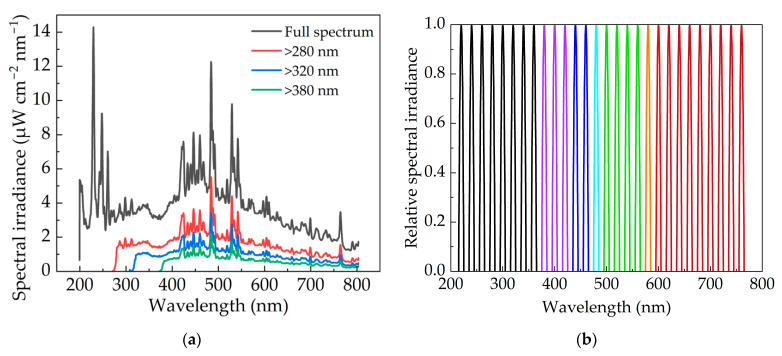
Spectral power distribution of the pulsed xenon lamp under (**a**) long-wave pass and (**b**) band-pass filters.

**Figure 9:**
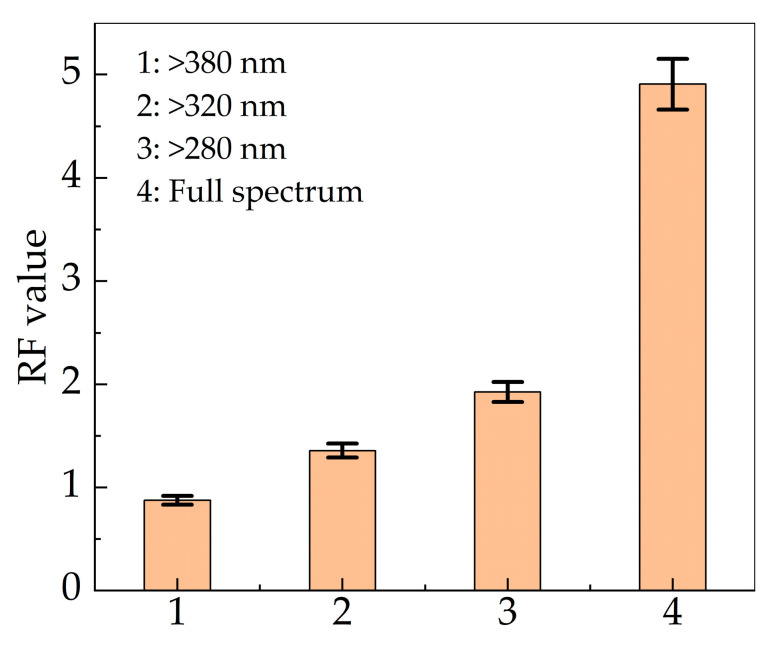
Sub-band RF values of pulsed xenon lamp.

In this paper, the influence of wavelength on inactivation efficiency was studied with the same irradiance. Various sub-band spectra of IPL for inactivation experiment are illustrated in Figure 8a, adjusted by long-wave pass filters. In addition, RF values are indicated in Figure 9. The inactivation efficiency of visible and infrared bands could be inspected by using a 380 nm long-wave pass (LWP) filter. The filter only retained the specific band, so the irradiance would be greatly reduced. The pulse width was set constant and the irradiance was 4.0 mW cm^−2^. When the irradiation dose was 0.5 mJ cm^−2^, the flash times measured 1250. The results showed that the RF value decreased significantly from 4.9072 to 0.8751 (Figure 9), which confirmed that ultraviolet radiation played a dominant role in the inactivation process of pulsed xenon lamp. Although visible and infrared bands demonstrated inactivation ability, the effect was very limited and unsuitable for using independently. Subsequently, UVA was added by using a 320 nm LWP filter, whereby the RF value increased by 0.4815. When UVB was added with a 280 nm LWP filter, the RF value further increased by 0.5677. The RF value of UVA and UVB was 1.0492, accounting for only 26.0% of the entire ultraviolet band. UVC irradiation was shown to be crucial for eliciting the bactericidal effect, which corresponded to the spectrum of the pulsed xenon lamp. According to the inactivation rate–wavelength curve of UV radiation [27] and the spectrum of the experimental pulsed xenon lamp, it can be calculated that the required doses of UVA and UVB are 0.8 and 4.5 mJ cm^−2^, respectively, to achieve the same RF value as UVC.

In order to further verify that the inactivation efficiency of the visible light was much lower than that of UV, band-pass filters of 400, 440, 480, 540, 580, 600 and 680 nm (bandwidth of 10 nm) were used to observe the inactivation efficiency of various mono-color light wavelengths. The spectra of the mono-color light wavelengths are plotted in Figure 8b, while the inactivation results are listed in Table 3. The RF value measured between 0.25–0.43 in the range from 400 nm to 680 nm, which proved that only the visible band of the pulsed xenon lamp could not meet the needs of inactivation scenarios. To achieve the inactivation requirement of an RF value > 3.0, the required dose is at least 29.4 mJ cm^−2^ [28] where only visible light of a specific wavelength is used.

**Figure 10:**
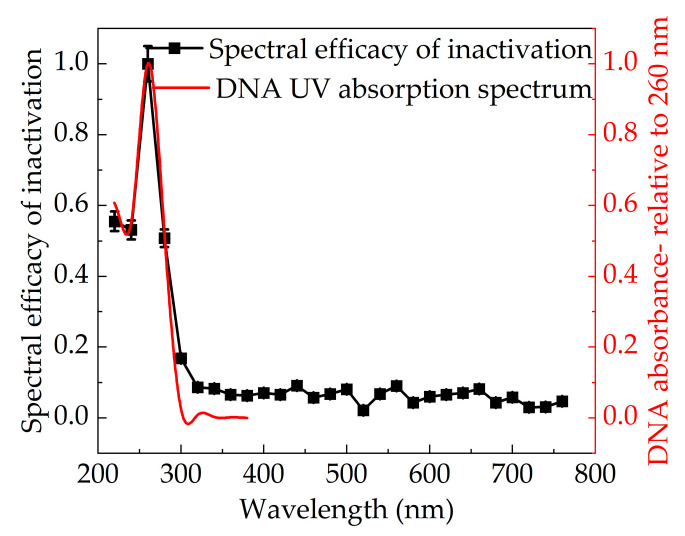
UV–visible spectral efficacy of pulsed xenon lamp inactivation.

Finally, 220–760 nm band-pass filters (bandwidth of 10 nm) with a gradient of 20 nm were used to determine the variation of UV–visible inactivation effects with wavelength. Figure 10 shows the spectral efficacy of inactivation of *Staphylococcus aureus*, which is calculated through the RF value of each mono band divided by the peak RF value. It could be found that the inactivation effect of mono light was first strengthened and then weakened with an increase in wavelength from 220 to 360 nm. The RF value displayed a maximum at 260 nm, with a population reduction of 9.4 log per mJ cm^−2^. The value of population reduction was higher than that in previous research works [21,25]. Firstly, the calculation method of irradiation dose in the study was defined according to the characteristics of pulsed light (Equation (6)). However, the dose was obtained by dividing the instantaneous irradiation energy (measured by the energy meter) by the irradiation area in other studies, which might not be exactly applicable to IPL. In addition, the irradiance was greater in the work, which had a significant role in promoting the inactivation process. Moreover, the maximum population reduction of the visible band was only 0.86 log per mJ cm^−2^ and far less than UV [21]. At the same time, it was obvious that the inactivation effect of UVC was better than that of UVB and UVC, and the corresponding RF value difference was more than 2, which is consistent with the sub-band experimental results. Furthermore, the RF value–wavelength curve resembles the UV absorption spectrum of DNA [26]. The absorbance of DNA was the highest at 260 nm, which corresponded to the strongest inactivation effect. At wavelengths greater than 300 nm, the absorbance of DNA was close to 0, and the corresponding RF value was less than 1, demonstrating that there was no obvious inactivation effect. The experimental results showed that genetic material destruction played a dominant role in the inactivation mechanism of pulsed light.

**Figure 11:**
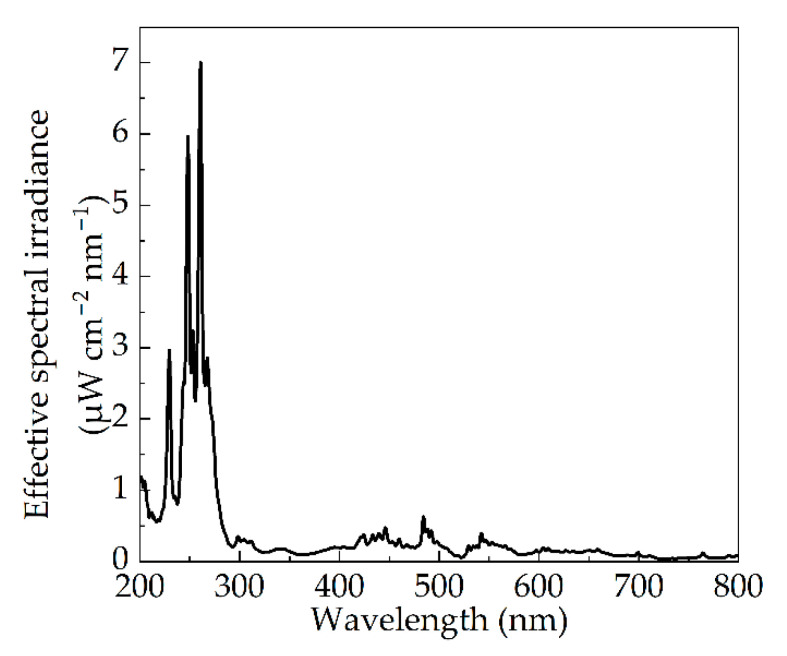
Effective spectral distribution of pulsed xenon lamp weighted by spectral inactivation curve in Figure 10.

Moreover, the spectral efficiency corresponding to each band-pass wavelength to the peak RF value (260 nm) of the whole band was calculated. The effective spectral irradiance of the pulsed xenon lamp is illustrated in Figure 11, which was obtained by multiplying together the spectral efficiency of inactivation and the spectral irradiance (Figure 3). It can be used to evaluate the effectiveness of pulsed xenon lamp spectrum for inactivation of *Staphylococcus aureus*. For example, although there was a section of strong spectral irradiance in the visible band as shown in Figure 3, the effective spectral irradiance in this range was even lower than that of UV, with lower spectral irradiance due to the low corresponding RF value. There is nothing to be gained in solely pursuing high spectral irradiance of visible band. In fact, a higher effective spectral irradiance can achieve a better inactivation effect. The IPL with greater UVC percentage is the better choice for microbial inactivation.

## 4. Conclusions

This study mainly investigated the effects of photoelectric parameters on the inactivation efficiency of a pulsed xenon lamp against *Staphylococcus aureus*. Increasing pulse width did not significantly improve the inactivation efficiency of *Staphylococcus aureus* without changing the irradiance and dose. Combined with the constant single-pulsed dose experiments and the comparison results of CW light and pulsed light, it was found that high irradiance was of great importance in enhancing the inactivation effect. In addition, the decisive position of genetic material destruction by UVC irradiation during the *Staphylococcus aureus* inactivation process was determined by measuring the inactivation efficiency corresponding to different wavelengths. According to the experimental results, a higher inactivation efficiency can be achieved when using a pulsed xenon lamp by increasing the radiance and proportion of UV irradiation, specifically UVC, among the full spectrum of wavelengths emitted by the lamp. 

## Figures and Tables

**Table 1:** Photoelectric parameters of pulsed xenon lamp under different pulse widths.

Pulse Width (μs)	*P*_sp_ (W)	*P*_max_ (kW)	*E*_1_ (mW cm^−2^)	*P*_r1_ (W)	*η* (%)	FWHM (μs)
40	10.5	292.8	4.3	5.5	52.4	40.0
60	13.0	297.6	5.1	6.6	50.8	60.0
80	24.9	316.8	9.9	12.6	50.6	80.0
100	28.9	316.8	11.4	14.6	50.5	100.0
120	32.4	316.8	13.1	16.8	51.9	120.0

**Table 2:** Percentage of each band spectral irradiance in the UV–visible spectrum of pulsed xenon lamp.

Band	Percentage in This Work	Percentage in Ref. [32]
UVC	15.1%	7.23%
UVB	5.41%	5.37%
UVA	9.99%	16.4%
UV	30.5%	29.0%
Visible	69.5%	71.0%

**Table 3:** RF values of various mono-color light wavelengths.

Wavelength (nm)	RF Value
400	0.39 ± 0.009
440	0.32 ± 0.012
480	0.25 ± 0.013
540	0.30 ± 0.013
580	0.28 ± 0.021
600	0.43 ± 0.018
680	0.34 ± 0.015

## Data Availability

The datasets generated and/or analyzed during the current study are available from the corresponding author on reasonable request.

## List of Symbols/Acronyms

**Symbol/Acronym**	**Interpretation**
IPL	Intense Pulse Light
RF	Reduction Factor
UV	Ultraviolet
CW	Continuous-wave
*u*(*t*)	Transient Voltage
*i*(*t*)	Transient Current
*p*(*t*)	Transient Electric Power
*P*_elec_	Effective Power
*T*	Pulse Period
*P*_sp_	Single-pulsed Electric Power
*f*	Frequency
FWHM	Full Width at Half Maxima
*P*_r1_	Radiant Flux (Radiant Power)
*E*_1_	Irradiance
*L*	Length of the Light Source
*D*	Distance
*α*	Half Apex Angle
*η*	Radiant Efficiency
*Q*	Irradiation Dose
*τ*	Optical Pulse Width
*N*	Flash Times
*Q*_1_	Single-pulsed Irradiation Dose
*N*_0_	Number of the Control Group
*N*_1_	Number of the Experimental Group
*μ*	Mean of Sample
*σ*	Standard Deviation of Sample
*P*_max_	Peak Power
CV	Coefficient of Variation
ΔRF	Difference of Reduction Factor
Δ*Q*	Difference of Dose
*E*	Average Irradiance Per Second
LWP	Long-Wave Pass
